# Effect of income on the cumulative incidence of COVID-19: an ecological study^*^

**DOI:** 10.1590/1518-8345.4475.3344

**Published:** 2020-06-26

**Authors:** Ricardo de Mattos Russo Rafael, Mercedes Neto, Davi Gomes Depret, Adriana Costa Gil, Mary Hellem Silva Fonseca, Reinaldo Souza-Santos

**Affiliations:** 1 Universidade do Estado do Rio de Janeiro, Faculdade de Enfermagem, Rio de Janeiro, RJ, Brazil.; 2 Scholarship holder at the Universidade do Estado do Rio de Janeiro, Centro de Estudos e Pesquisas em Saúde Coletiva (CEPESC), Rio de Janeiro, RJ, Brazil.; 3 Scholarship holder at the Coordenação de Aperfeiçoamento de Pessoal de Nível Superior (CAPES), Brazil.; 4 Fundação Oswaldo Cruz, Escola Nacional de Saúde Pública Sergio Arouca, Rio de Janeiro, RJ, Brazil.

**Keywords:** Coronavirus, Pandemics, Uses of Epidemiology, Income, Socioeconomic Factors, Incidence, Coronavírus, Pandemias, Aplicações da Epidemiologia, Renda, Fatores Socioeconômicos, Incidência, Coronavirus, Pandemias, Usos de la Epidemiología, Renta, Factores Socioeconómicos, Incidencia

## Abstract

**Objective:**

to analyze the relationship between *per capita* income and the cumulative incidence of COVID-19 in the neighborhoods of the city of Rio de Janeiro, RJ, Brazil.

**Method:**

an ecological study using neighborhoods as units of analysis. The cumulative incidence rate *per* 100,000 inhabitants and the median of potential confounding variables (sex, race, and age) were calculated. Multiple analysis included quantile regression, estimating the regression coefficients of the variable income for every five percentiles from the 10^th^ to 90^th^ percentiles to verify the relationship between income and incidence.

**Results:**

the city’s rate was 36.58 new cases *per* 100,000 inhabitants. In general, the highest rates were observed in the wealthiest regions. Multiple analysis was consistent with this observation since the *per capita* income affected all percentiles analyzed, with a median regression coefficient of 0.02 (p-value <0.001; R^2^ 32.93). That is, there is an increase of R$ 0.02 in the neighborhood’s *per capita* income for every unit of incidence.

**Conclusion:**

cumulative incident rates of COVID-19 are influenced by one’s neighborhood of residency, suggesting that access to testing is uneven.

## Abstract (pt)

**Objetivo:**

analisar a relação entre renda *per capita* e incidência acumulada de COVID-19 nos bairros do município do Rio de Janeiro.

**Método:**

estudo ecológico utilizando os bairros como unidades de análise. Foram calculadas as taxas de incidência acumulada por 100 mil habitantes e a mediana de possíveis variáveis confundidoras (sexo, raça/cor e idade). A análise múltipla contou com técnica de regressão quantílica, estimando-se os coeficientes de regressão da variável renda a cada cinco percentis no intervalo entre os percentis dez e 90, a fim de verificar a relação entre renda e incidência.

**Resultados:**

a taxa municipal foi de 36,58 casos novos por 100 mil habitantes. Em geral, as maiores taxas da doença foram observadas nas regiões de elevada renda. A análise múltipla coadunou com esta observação, já que a renda *per capita* apresentou efeito em todos os percentis analisados, tendo coeficiente de regressão mediano de 0,02 (p-valor <0,001; R^2^ 32,93), ou seja, que a cada unidade de incidência elevam-se R$0,02 na renda *per capita* do bairro.

**Conclusão:**

as taxas de incidência acumulada de COVID-19 são influenciadas pela renda do bairro de residência dos casos, sugerindo que o acesso aos exames esteja ocorrendo de modo desigual.

## Abstract (es)

**Objetivo:**

analizar la relación entre la renta per cápita y la incidencia acumulada de COVID-19 en los barrios del municipio de Rio de Janeiro.

**Método:**

estudio ecológico utilizando los barrios como unidades de análisis. Fueron calculadas las tasas de incidencia acumuladas por cada 100 000 habitantes y la mediana de las posibles variables mezcladas (sexo, raza/color y edad). El análisis múltiple contó con la técnica de regresión cuantílica, estimándose los coeficientes de regresión de la variable renta por cada cinco percentiles en el intervalo entre los percentiles 10 y 90, con el fin de verificar la relación entre la renta alquiler y la incidencia.

**Resultados:**

la tasa municipal fue de 36,58 casos nuevos por 100 000 habitantes. En general, se observaron las mayores tasas de la enfermedad en las regiones de renta alta. El análisis múltiple coincidió con esta observación, ya que la renta per cápita tuvo efecto en todos los percentiles analizados, con un coeficiente de regresión medio de 0,02 (valor de p <0,001; R^2^ 32,93). Es decir, para cada unidad de incidencia aumenta R$0,02 la renta per cápita del barrio.

**Conclusión:**

las tasas de incidencia acumuladas de COVID-19 se ven influidas por la renta del barrio de residencia de los casos, lo que sugiere que el acceso a los exámenes se produce de forma desigual.

## Introduction

With the declaration of the new Coronavirus disease (COVID-19) pandemic by the World Health Organization on March 11^th^, 2020, the escalation of studies addressing potential strategies to cope with the disease has reached a new level^(1-2)^. A COVID-19 vaccine, which is the primary means to decrease the population’s susceptibility, and consequently the best way to flatten epidemic curves, is inexistent up to now. Thus, only measures to intervene in the social structure as a way to reduce the speed at which COVID-19 spreads remain^(3)^.

The need to identify the more vulnerable groups and the disease’s behavior has gained space in the editorials of the leading international and Brazilian periodicals. Studies addressing early diagnosis, treatment, and containment mechanisms have guided debates worldwide^(4-5)^. Much uncertainty remains though regarding the disease’s social dynamics in developing countries and more impoverished regions. The reason is that accumulated theoretical knowledge regarding infectious diseases indicates that there is a potential relationship between per capita income and the incidence of the disease; its spread dynamics are different in European and North American regions, where a significant portion of studies addressing COVID-19 originated^(6)^. Studies addressing HIV, tuberculosis, and leprosy have already reported this relationship so that it remains to be known how this variable behaves in the case of the COVID-19 pandemic, a critical gap so far^(7-9)^.

Based on the international debate on the topic, this study postulates three other factors related to income, mainly due to their possible role as confounding variables in analytical models. The first postulate found in the Brazilian literature is that *per capita* income is related to race. Economic and social inequalities produce harmful effects on illness and access to health services^(10)^. To some extent, this relationship can be reproduced in the COVID-19 context, so that these effects need to be investigated in analytical models. There is abundant literature reporting that women more frequently access health services, either because of the way they conceive the health-disease construct and understand their self-assessment of health, or because of the – already agreed upon - harmful effects of the traditional view of gender on the male population^(11-12)^. In this sense, the distribution of sex in the composition of the population may somehow affect the epidemic’s behavior.

Bringing up this debate in which race and socioeconomic class are intimately involved in the production of health and disease, an American newspaper presents a report on the possibility of more significant contagion and lethality among Afro-descendent individuals^(13)^. If we consider that the relationship of income distribution in Brazil is affected by race, investigations are needed to address these variables in explanatory models.

Finally, similar to other respiratory infections, advanced age (the elderly) has been appointed as the variable leading to the most severe manifestations of the disease in most papers investigating the COVID-19 pandemic^(14-15)^. In this sense, it seems essential to control models intended to investigate the relationship between income and COVID-19 for sex, race, and age, to perform a more complex explanatory analysis, an aspect that is lacking in the knowledge acquired so far.

Therefore, this study presents an alternative hypothesis, that the cumulative incidence rates are directly linked to the *per capita* income of neighborhoods, regardless of the remaining predictors (composition according to sex, age, and race). Therefore, this study’s objective is: to analyze the relationship between *per capita* income and cumulative incidence of the COVID-19 in the neighborhoods in the city of Rio de Janeiro, RJ, Brazil.

## Method

This is an ecological study, the units of analysis of which were 159 neighborhoods in the city of Rio de Janeiro, located in the metropolitan region of Rio de Janeiro. The option to investigate at a neighborhood level, especially in the city of Rio de Janeiro, is because of the possibility to understand social nuances and inequalities, which may ultimately behave similarly to other Brazilian regions.

The databases made available by the Pereira Passos Institute (IPP) and the government of the state of Rio de Janeiro were used. The variables concerning the population structure were collected through the Data Rio application of the IPP, using data from the 2010 Census provided by the Instituto Brasileiro de Geografia e Estatística (Brazilian Institute of Geography and Statistics)^(16)^. They are: population, distribution of the population according to sex, age distribution, self-reported race, and *per capit*a income. The neighborhood was the unit of initial observation. Afterward, data were aggregated according to Administrative Regions.

The databases of the government of the state of Rio de Janeiro that were available to the public were used in data collection, specifically through the Coronavírus COVID-19^(17)^ application. The variables collected in this application were: sex, age, date of report, city of residence, neighborhood, and test result. Two aspects need to be clarified: 1) the unit of observation used by the state government system is the individual; 2) the report of the number of cases, which is updated daily, is only available after laboratory confirmation of positive testing – information regarding suspected cases discarded upon laboratory exam is not disclosed.

Data were collected from April 7^th^ to 13^th^ 2020. Considering the speed at which data are produced due to the progression phase of the COVID-19 epidemic in Brazil, it is important to note that data concerning the disease occurred on April 13^th^, totaling 2,323 observations. Of these, 11 were excluded: nine because they referred to another city, and two were imported cases (allochthonous). This database contains community transmissions from April 5^th^ to 13^th^, 2020. After data collection, variables were aggregated *per* neighborhood and administrative region, to facilitate the presentation of results.

This study’s outcome variable, COVID-19 Cumulative Incidence Rate, is obtained by dividing the cumulative number of confirmed cases since the population in the neighborhood started being observed, using 100,000 as the basis of the indicator. Thus, the results of this variable were expressed in new cases *per* 100,000 inhabitants. The exposure variable of interest, *per capita* income, was collected and used in the analysis in its original format.

Independent control variables were considered according to the theoretical model presented in the introduction: the percentage of women in the population, the proportion of elderly individuals above the age of 60, and the percentage of afro-descendent and mixed-race individuals. These variables were constructed by dividing the numerator, where the interest information was placed (e.g., the number of women in the population) by the neighborhood’s population. The results were multiplied by 100.

The building, cleansing, and statistical processing of the databases were performed using Stata SE 15 (StataCorp LP, College Station, United States). First, univariate analysis was performed to apply descriptive analyses, and then bivariate analysis and multiple quantile regression were performed to identify the dependence and effect of the *per capita* income and COVID-١٩ incidence rate.

Because the outcome and exposure variables are two continuous numerical variables, the Shapiro-Wilk test was used to test whether the distribution was normal. The test considered the null hypothesis that the distribution is normal and close to a Gaussian curve. The Shapiro-Wilk test for the variables Incidence Rate and *Per capita* Income presented a Z statistic of 8.63 and 828, respectively. In both cases, the p-value was below 0.001. In addition to this test, the authors also performed visual tests of distribution, confirming that the distribution of data was not normal. Therefore, the authors needed to propose models of analysis that considered the characteristics of this distribution.

Hence, due to the non-normal distribution, the univariate analyses consisted of calculating the median values of the percentages of women, of those 60 years or older, and afro-descendent individuals, and *per capita* income in Reais (R$) aggregated in administrative regions, according to criteria established by the city of Rio de Janeiro^(18)^.

A bivariate analysis was performed to measure the association between the exposure variable of interest and outcome, using Spearman’s correlation because the distribution was not normal. This test considered the null hypothesis that two variables are independent, considered to be statistically significant at 5% (type 1 error).

The effect of the exposure variable on the outcome was estimated using a multiple quantile regression analysis, using the neighborhood as the unit of analysis^(19)^. This regression model was chosen because the distribution was not normal; the outcome variable was skewed to the left; and there was high heteroscedasticity in the relationship between the outcome and exposure of interest variables, observed by the Breusch-Pagan & Cook-Weisberg test (X^2^ 857.89; p-valor: <0.001). This effect makes certain percentiles be influenced by the model predictors, while linear regression models based on the least squares method are not appropriate. In this sense, although not very widespread in the health field, including epidemiology, quantile regression, an already traditional method in econometric models, is a methodological option.

The regression coefficients were estimated in this multiple analysis for every five percentiles from the 10^th^ to 90^th^ percentiles, using the *per capita* income variable as the exposure of interest and forcing the entry of the remaining independent variables, always from the same regression model and according to this study’s theoretical model. The standard error was calculated using the bootstrap replication technique with 20 repetitions. Standard error was calculated using the bootstrap technique with 20 repetitions. Taking as reference the null hypothesis that the effect of the exposure variable is equal in all the percentiles, the Wald test was calculated using a reference error of 5%.

Finally, considering this study presents secondary data analysis and uses databases widely accessible in public sites, ethical recommendations for studies addressing human subjects do not apply, and this study is exempted from the opinion of a Research Ethics Committee.

## Results

Table 1 presents the characterization of the neighborhoods in the city of Rio de Janeiro, aggregated by administrative regions, according to the variables: number of neighborhoods in the region and the median percentage of women, of individuals aged 60 years old or older, of Afro-descendent individuals, and *per capita* income (R$). Attention is drawn to the fact that Copacabana, Tijuca, and Botafogo rank first in terms of median percentages of women and individuals aged 60 years old or older, and rank last in terms of the median percentage of Afro-descendent individuals. At the opposite end is Complexo do Alemão, presenting the lowest *per capita* income in the city and the lowest percentage of women and elderly individuals. The region also presents one of the largest Afro-descendent populations in the city, second only to Cidade de Deus.

**Table 1:** – Characterization of the neighborhoods in the city of Rio de Janeiro, aggregated by administrative regions, according to the variables: number of neighborhoods in the region and the median percentages of women, of individuals 60 years old or older, of Afro-descendent individuals, and *per capita* income (R$). Rio de Janeiro, RJ, Brazil, 2020

Administrative region	Number of neighborhoods	% Women	% ≥ 60 year- old individuals	% Afro-descendent individuals	*Per capita* income(R$)*
Anchieta	4	52.92	14.28	58.64	672.29
Bangu	4	52.47	12.10	63.60	653.26
Barra da Tijuca	8	50.85	7.89	51.97	1387.77
Botafogo	8	55.63	22.36	18.69	3886.05
Campo Grande	5	51.86	10.76	54.37	613.97
Centro	1	53.12	21.02	40.37	1533.38
Cidade de Deus	1	52.93	10.41	72.14	517.99
Comp. do Alemão	1	51.12	8.04	65.85	390.93
Copacabana	2	56.60	27.48	15.89	3821.18
Guaratiba	3	51.15	14.43	59.54	556.62
Ilha do Governador	15	53.26	15.23	48.25	1232.26
Inhaúma	7	53.86	15.26	48.05	1090.60
Irajá	6	54.77	16.36	41.56	993.62
Jacarepaguá	10	53.25	14.87	51.33	1154.20
Lagoa	7	55.47	22.04	46.82	6098.88
Madureira	13	54.13	15.38	52.71	788.18
Maré	1	50.88	6.96	19.96	456.72
Méier	15	54.51	17.97	41.43	1122.58
Paquetá	1	51.53	23.15	56.04	1011.52
Pavuna	6	52.23	11.35	62.13	560.38
Penha	6	52.41	14.60	52.80	774.89
Portuária	4	51.62	11.38	56.47	505.50
Ramos	4	53.86	17.96	42.69	995.38
Realengo	6	52.95	13.07	49.65	1279.35
Rio Comprido	4	53.39	14.76	54.42	869.69
Rocinha	1	50.67	5.62	55.60	455.67
Santa Cruz	3	51.96	10.07	53.87	509.71
Santa Teresa	1	52.97	13.13	65.69	1281.08
São Cristóvão	4	51.99	13.01	49.99	577.60
Tijuca	3	56.31	22.44	36.94	3023.42
Vigário Geral	1	52.25	11.00	63.26	508.27
Vila Isabel	4	55.77	20.81	33.54	2362.79

^*^Minimum wage R$ 510.00, Brazil, 2010

The COVID-19 Cumulative Incidence Rate in the city was 36.58 new cases *per* 100,000 inhabitants, totaling 2,312 new cases confirmed in the period. Of these, 599 cases did not provide information regarding the neighborhood of residence (34.74%) while 146 (11.83%) did not provide information on age, and were excluded. Table 2 presents the rates aggregated by administrative region in the general population, according to women, men, individuals aged 60 years old or older, and the non-elderly population (<60 years old). Note that the Lagoa region leads with the highest incidence in all the variables analyzed, followed by Tijuca and Copacabana. Ilha de Paquetá and Jacarezinho, on the other hand, did not present cases so far. Maré and Complexo do Alemão present 1.08 and 1.45 new cases *per* 100,000 inhabitants, respectively. Specifically, concerning the incidence rate among 60-year-old or older individuals, Rocinha presents the highest result.

**Table 2:** – COVID-19 Cumulative Incidence Rate *per* 100,000 inhabitants in the city’s administrative regions. Rio de Janeiro. RJ. Brazil. 2020

Administrative Region	General population	Women	Men	≥60-year-old individuals	<60-year-old individuals
Anchieta	15.16	18.87	11.89	4.52	14.53
Bangu	12.62	14.87	10.48	24.85	9.58
Barra da Tijuca	58.51	61.75	55.52	124.23	41.89
Botafogo	66.74	74.93	60.31	70.04	50.47
Campo Grande	13.65	18.60	9.15	25.25	10.70
Centro	58.33	51.84	64.06	127.18	31.60
Cidade de Deus	13.69	23.27	5.17	0.00	13.69
Complexo do Alemão	1.45	2.96	0.00	0.00	1.45
Copacabana	78.17	87.03	71.55	86.91	52.73
Guaratiba	4.87	8.32	1.59	0.00	4.87
Ilha de Paquetá	-	-	-	-	-
Ilha do Governador	18.82	21.01	16.87	21.62	15.52
Inhaúma	26.80	32.28	22.10	47.93	19.35
Irajá	29.56	32.38	27.20	48.28	21.19
Jacarepaguá	17.11	19.15	15.28	37.55	12.40
Jacarezinho	-	-	-	-	-
Lagoa	732.05	723.84	739.68	279.09	97.75
Madureira	37.55	54.64	23.72	26.65	12.64
Maré	1.08	1.17	1.00	11.08	2.31
Méier	69.35	83.15	56.04	43.43	14.58
Pavuna	8.55	13.83	4.15	29.63	12.93
Penha	11.01	14.18	8.18	17.91	9.69
Portuária	2.69	1.13	4.12	18.94	8.22
Ramos	51.37	55.47	47.56	16.83	13.71
Realengo	23.50	26.70	20.73	33.77	10.29
Rio Comprido	9.05	8.75	9.32	34.52	22.79
Rocinha	43.05	46.51	40.07	153.81	40.37
Santa Cruz	27.39	35.08	19.92	2.64	4.88
Santa Teresa	2.17	1.69	2.61	18.61	17.10
São Cristóvão	48.87	77.93	23.06	45.87	17.67
Tijuca	96.58	114.09	80.07	55.69	31.90
Vigário Geral	17.05	21.31	13.72	28.31	19.09
Vila Isabel	49.20	58.46	40.75	56.53	23.77

Rejecting the null hypothesis that the two variables are independent, Spearman’s test resulted in Rho 0.524; with a p-value <0.001. Table 3 presents the quantile regression results. Note that *per capita* income presented an effect in all the strata analyzes. The β coefficient of the *per capita* income variable also increases according to the percentile, indicating this variable presents higher explanatory power in the model. Note that the adjusted R^2^ increases as a function of the Incidence Rate percentile, while the number of predictors with a statistically significant effect, decreases.

**Table 3:** – Multiple quantile regression analysis between the COVID-19 cumulative incident rates, *per capita* income, and percentages of individuals aged 60 years old or older, Afro-descendent individuals, and women in the neighborhoods in the city of Rio de Janeiro, RJ, Brazil, 2020

Predictors	Adjusted R^2^	β*	CI 95%^†^		T^||^	p-value
			LI^‡^	LS^§^		
Percentile 10	23.41					
*Per capita* income^¶^		0.01	0.01	0.02	3.6	<0.001
% 60-year-old or older individuals		0.67	-1.02	2.36	0.78	0.435
% Afro-descendent individuals		0.10	-0.17	0.38	0.76	0.448
% Women		-1.31	-5.65	3.04	-0.59	0.553
Percentile 25	26.32					
*Per capita* income^¶^		0.02	0.01	0.02	7.5	<0.001
% 60-year-old or older individuals		-0.23	-2.02	1.56	-0.26	0.798
% Afro-descendent individuals		0.06	-0.08	0.20	0.84	0.403
% Women		-0.06	-5.26	5.15	-0.02	0.983
Percentile 50	32.93					
*Per capita* income^¶^		0.02	0.01	0.03	6.09	<0.001
% 60-year-old or older individuals		1.24	-0.65	3.12	1.3	0.197
% Afro-descendent individuals		0.27	-0.01	0.56	1.88	0.062
% Women		-5.01	-10.64	0.63	-1.76	0.081
Percentile 75	41.93					
*Per capita* income^¶^		0.03	0.02	0.04	5.1	<0.001
% 60-year-old or older individuals		1.30	-1.38	3.99	0.96	0.339
% Afro-descendent individuals		0.37	-0.14	0.88	1.43	0.155
% Women		-5.61	-13.84	2.62	-1.35	0.180
Percentile 90	50.88					
*Per capita* income^¶^		0.03	0.01	0.05	3.30	0.001
% 60-year-old or older individuals		4.30	-0.75	9.35	1.68	0.094
% Afro-descendent individuals		0.29	-0.67	1.24	0.59	0.554
% Women		-19.88	-34.26	-5.50	-2.73	0.007

*Regression coefficient; ^†^Confidence Interval 95%; ^‡^Lower limit; ^§^Upper limit; ^||^T statistics; ^¶^Minimum wage R$ 510.00, Brazil, 2010

**Figure 1:**
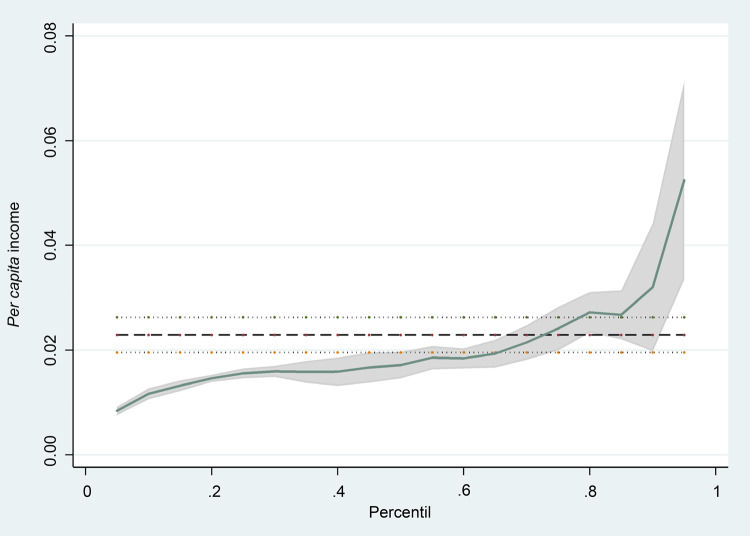
– The quantile regression coefficients of the *per capita* income for the percentiles of the COVID-19 cumulative incidence rate adjusted for the percentages of individuals aged 60 years old or older, Afro-descendent and women in the neighborhoods of the city of Rio de Janeiro, RJ, Brazil, 2020

Figure 1 presents a β adjusted coefficient of the *per capita* income variable, according to the percentile analyzed. Note the accentuated effect of the variable in the last percentile. Rejecting the null hypothesis that the adjusted *per capita* income variable is the same in all the percentiles tested, the F statistic was equal to 4.18, with a p-value equal to 0.003.

## Discussion

Relating income with the incidence of infectious diseases is not new, as this relationship has been reported by other studies addressing infectious diseases^(7-9,20)^. Since the beginning of the pandemic, health authorities have expressed concern with the behavior of the epidemic in the world’s most impoverished regions^(6,21-22)^. Considering knowledge of the epidemiological chain of other infectious diseases, many situations may determine a different behavior of COVID-19 in these places. These situations include greater spread due to the high population and household density, interaction with other chronic and infectious diseases coupled with the health systems’ diminished capacity to provide treatment, and greater lethality due to the decreased capacity of intensive care units^(23)^.

This study’s main finding indicates that *per capita* income has a significant effect on the cumulative incidence rate of COVID-19, progressively increasing its influence according to the increase in the percentiles. Note that, in the groups with the lowest incidence, each incidence unit is related to an increase of R$ 0.01 in income, while this increase is of R$0.03 in the 75^th^ and 90^th^ percentiles. In this sense, more affluent neighborhoods tend to present higher rates, regardless of the percentage of elderly individuals, Afro-descendants, and women.

From what is known about the social structures in the city of Rio de Janeiro, considering a higher population density on the outskirts and more impoverished neighborhoods, the natural hypothesis is that these regions would experience a greater spread of the disease, as it occurs in the epidemiological chain of other respiratory diseases^(7,9,20)^. Nonetheless, the practical effect of these findings gains strength in the observation that the wealthiest regions such as Lagoa, Tijuca and Copacabana, present high cumulative incidence rates, occupying the first of the five ranks of *per capita* income. On the other hand, Maré and Complexo do Alemão, which rank last in terms of income, also present the lowest incidence rates of the disease.

This paradox was recently raised in a technical document reported by the Federal Rural University of Rio de Janeiro (UFRRJ) regarding the COVID-19 sociospatial dynamics in the program areas of Rio de Janeiro and Baixada Fluminense^(24)^. The results reported by this document indicate a higher number of cases in regions with the highest economic power, contradicting the distribution typically observed in Brazilian states. The document presents two potential explanations: 1) that the social isolation adopted in the wealthiest neighborhoods may positively affect the spread chain in the more impoverished neighborhoods; and 2) that underreporting rates are higher in poorer regions.

In addition to these explanations, it is important to note that the media widely reported that the first cases imported into the city were among residents from the South of the city and Barra da Tijuca, who had recently returned from Europe. Hence, the virus started spreading mainly in these areas, which does not exclude the remaining hypotheses under analysis.

The Oswaldo Cruz Foundation, monitoring the COVID-19 and using Big Data from *Instituto de Comunicação e Informação Científica e Tecnológica em Saúde* [Institute of Scientific and Technological Communication and Information in Health], launched the MonitoraCovid-19. The system monitors variables concerning the movement of people, using it as a proxy in the assessment of social isolation. The system’s results show decreased use of public transportation in the city compared to the period before the pandemic, especially when the first legal measures were adopted to prevent the spread of the disease^(25)^. On the other hand, the results also show certain instability in the flow of people over the days, especially on the weekends. It is not, however, possible to state that groups do not intercommunicate, especially between neighborhoods in a time when people are experiencing the increasing precariousness of the workforce^(26)^.

Considering that the largest populations with the worst access to services are concentrated in the most impoverished regions, we conclude that the disease is spreading more rapidly in the regions with the lowest *per capita* income, even though it was initiated in the areas with the highest *per capita* income^(27)^. For these reasons, it seems unlikely that the relationship between income and incidence is a reflex of the social isolation adopted in the city’s wealthiest regions. On the contrary, the explanation likely lies in the second hypothesis raised in the UFRRJ report, that is, high underreporting rates in the poorest regions are the origin of this phenomenon.

The key to solving this theoretical *imbroglio* may be linked to three interrelated aspects: classification, testing, and unequal access to health services. The information provided by the health authorities concerning COVID-19 cases does not include suspected and probable cases in the calculation of databases, recommending social isolation without proper testing. The Brazilian government has recommended in various official documents that mild and asymptomatic cases remain at home in isolation, without, however, requiring screening testing. The important selection bias expressed in the option of presenting indicators based only on confirmed cases indicates a considerable and fallacious decrease in the incidence of the disease, preventing the planning of more effective public policies^(3)^.

There is a consensus on the expressive underreporting of the disease. A mathematical model produced by the Imperial College London indicates a mean detection rate well below the actual context of the pandemic^(28)^. The number of cases in Brazil is estimated to be 12 to 15 times greater than that reported by the Ministry of Health^(29-30)^.

One study based on the Chinese experience in Wuhan estimates that 86% of the infectious cases were not documented before the country’s health authorities imposed restricted mobility, and these infectious cases were the source of contamination of approximately 80% of new cases^(31)^. Even though there is no consensus regarding this matter, a recently published study reports that the disease may continue to spread 8 days after the symptoms cease, highlighting the need to record suspected and probable cases^(32)^. Ultimately, it means that a lack of proper documentation of cases directly affects the adoption of preventive measures and other sanitary barriers to contain the epidemic, which directly contributes to worsening the epidemic.

A lack of public information regarding the number of COVID-19 tests and how they are distributed among states and in the interior of each city is also another problem to be faced in the context of the pandemic in Brazil. It is known that Brazil is among the countries that least perform screening testing, which decreases its ability to map cases based on suspected, probable, and contact cases^(33)^. Brazil performs 296 tests *per* 1 million inhabitants while the United States, Spain, and Italy test 10,266, 19,896, and 19,490, respectively.

Additionally, it is important to note that, due to a lack of rapid tests in most health care units, COVID-19 testing has taken place in specialized laboratories, clinics, and hospitals. Specifically in Rio de Janeiro, social inequality and poor access to health services are probably contributing to the higher cumulative incidence in the wealthiest regions, especially in downtown and the south, with the highest *per capita* income and the largest number of public and private facilities. That is, the explanatory hypothesis is that the incident rate is not truly low in the more impoverished regions, but that there is rather a lower capacity to detect the diseases in these locations.

A similar aspect was observed in a study addressing Brazilian cities’ profile and the presence of drug-resistant tuberculosis. The worst results of this form of the disease were associated with a more abundant supply of culture tests and better economic indicators, among which income^(7)^. Spatial analysis of the relationship between HIV infection and social determinants presented results that are consistent with this study’s findings, where the highest infection rates are present in places with improved living conditions, reinforcing the idea that unequal access to screening tests of infectious diseases directly affects the report of diseases^(8)^.

Therefore, it is expected that approximately 5% of the infected people will need intensive care, 2.3% will require mechanical ventilation, while Brazil presents problems in the number and distribution of intensive care beds. Thus, it is urgent to expand COVID-19 testing in the population, decreasing regional inequalities in Brazilian cities^(34)^. It is worth mentioning that even in China, where the health system was minimally organized to receive COVID-19 patients, the influence of disparities in the access to health services was observed^(35)^. After all, capital-based necropolitics seem to be disseminated to the point of reaching poverty and the lives of poor people worldwide.

Despite this study’s relevant findings, it is important to interpret these results considering its limitations. The low quality of data, verified by the significant percentage of cases without identifying the individuals’ housing neighborhood, and the partiality of the cases tested, can produce uncertainty in the calculation of incidences. The format used by the Brazilian Ministry of Health and the government of Rio de Janeiro to disclose data is another aspect that hinders more in-depth analyses. Although within a unit of analysis, such as a neighborhood, may coexist distinct and unequal social conditions, this is the smallest unit of analysis possible to achieve with the format used by the Brazilian government. As previously mentioned, to decrease the influence of heterogeneity of data, this study considered multiple analysis techniques that addressed such disparities, as is the case of quantile regression.

The option to use the 2010 Census as the population base can also produce a discreet increase in the incident rates, considering that the denominator is undersized. However, even if losing its validity, this choice was made to facilitate comparison of results. At the limit, the interpretation of incidence rates could be even lower, which would constitute more considerable underreporting of COVID-19 cases. Therefore, this study represents an advancement in the analysis of this phenomenon, mainly because it considers that current investigations have contemplated states and cities, without investigating differences existing within these places.

## Conclusion

This study’s results indicate the hypothesis that the disease’s incidence rates in the city of Rio de Janeiro are related to *per capita* income, regardless of other predictors. Considering the low testing in Brazil, and consequent underreporting, already reported by other studies, these results indicate that COVID-19 testing is more widely disseminated in the wealthiest regions of the city. Potentially unequal access among suspected cases and the functionalist role of capital-based necropolitics should be considered in future analyses addressing this matter, primarily because of the need to better document cases of the disease, decreasing potential inequalities in the access to health services and designing better public policies to deal with the pandemic.
